# Application of Response Surface-Corrected Finite Element Model and Bayesian Neural Networks to Predict the Dynamic Response of Forth Road Bridges under Strong Winds

**DOI:** 10.3390/s24072091

**Published:** 2024-03-25

**Authors:** Yan Liu, Xiaolin Meng, Liangliang Hu, Yan Bao, Craig Hancock

**Affiliations:** 1 The Key Laboratory of Urban Security and Disaster Engineering of the Ministry of Education, Beijing University of Technology, Beijing 100124, China; liuyan0stone@emails.bjut.edu.cn (Y.L.); huliangliang@emails.bjut.edu.cn (L.H.); baoy@bjut.edu.cn (Y.B.); 2 School of Architecture, Building and Civil Engineering, Loughborough University, Loughborough LE11 3TU, UK; c.m.hancock@lboro.ac.uk

**Keywords:** digital twin, displacement prediction, structural health monitoring, large-span bridge structure, strong winds

## Abstract

With the rapid development of big data, the Internet of Things (IoT), and other technological advancements, digital twin (DT) technology is increasingly being applied to the field of bridge structural health monitoring. Achieving the precise implementation of DT relies significantly on a dual-drive approach, combining the influence of both physical model-driven and data-driven methodologies. In this paper, two methods are proposed to predict the displacement and dynamic response of structures under strong winds, namely, a Bayesian Neural Network (BNN) model based on Bayesian inference and a finite element model (FEM) method modified based on genetic algorithms (GAs) and multi-objective optimization (MOO) using response surface methodology (RSM). The characteristics of these approaches in predicting the dynamic response of large-span bridges are explored, and a comparative analysis is conducted to evaluate their differences in computational accuracy, efficiency, model complexity, interpretability, and comprehensiveness. The characteristics of the two methods were evaluated using data collected on the Forth Road Bridge (FRB) as an example under unusual weather conditions with strong wind action. This work proposes a dual-driven approach, integrating machine learning and FEM with GNSS and Earth Observation for Structural Health Monitoring (GeoSHM), to bridge the gap in the limited application of dual-driven methods primarily applied for small- and medium-sized bridges to large-span bridge structures. The research results show that the BNN model achieved higher $R^{2}$ values for predicting the Y and Z displacements (0.9073 and 0.7969, respectively) compared to the FEM model (0.6167 and 0.6283). The BNN model exhibited significantly faster computation, taking only 20 s, while the FEM model required 5 h. However, the physical model provided higher interpretability and the ability to predict the dynamic response of the entire structure. These findings help to promote the further integration of these two approaches to obtain an accurate and comprehensive dual-driven approach for predicting the structural dynamic response of large-span bridge structures affected by strong wind loading.

## 1. Introduction

Throughout the last century, the construction of large bridges spanning rivers, lakes, and oceans has played a crucial role in facilitating global economic activities and the smooth operation of transportation systems. However, it is well known that long-span bridges, especially suspension bridges, are susceptible to large structural displacements and local damage under extreme weather conditions such as strong winds and high temperature variations, which can potentially affect their overall stability and serviceability. Therefore, the accurate prediction of the dynamic response of large-span bridge structures under extreme weather conditions is essential for the health monitoring of bridge structures.

Sun et al. [1] proposed that traditional approaches to bridge analysis using monitoring data can be categorized into model-based and data-driven approaches. Model-based approaches involve the use of finite element analysis to construct an accurate finite element model (FEM) of bridges by updating model parameters using input–output measurements [2]. These models are then used to predict the dynamic responses of bridge structures under various load conditions, including vehicle and environmental loads. Many studies have focused on analyzing the dynamic characteristics of bridge structures, such as natural frequencies, damping, and free vibration characteristics, using finite element analysis (FEA) [3]. With increasing bridge spans, the flexibility of these structures increases, resulting in decreased vibration frequencies and damping, making them more susceptible to wind loading [4,5]. Under the influence of wind loading, the nonlinear behavior of large-span bridges becomes more prominent, particularly in extreme weather conditions such as strong winds or typhoons, leading to significant displacements and deformations [6]. When affected by wind loading, bridge structures may experience large displacements, which not only affect the comfort and safety of vehicles crossing the bridge but also have adverse effects on the structures themselves [7]. Strong winds can induce significant displacement responses, reduce structural stiffness, and potentially lead to structural damage. Therefore, many studies have examined the aerostatic stability of large-span bridge structures under wind loads, taking into account factors such as static non-oscillatory dispersion and torsional dispersion and investigating the relationship between critical wind speed and bridge displacement response [8,9]. Several studies have been conducted to predict the nonlinear displacement response of bridges under wind loading [10,11]. Hong et al. [12] developed a three-dimensional finite element model of a suspension bridge with geometric nonlinearities and predicted the time-domain dynamic response to wind loads using a full-bridge aeroelastic simulation. They conducted a case study of a suspension bridge with a main span of 728 m for validation, and the average error in the predicted and measured mid-span vertical displacements was 19%, while the error in mid-span lateral displacements was about 48%. One of the important reasons for this poor accuracy is the discrepancy between the wind field structures in the full-bridge aeroelastic simulation and the actual location of the bridge structure. Wang et al. [13] proposed a model that accounts for the stochastic nature of traffic flow and applied it to the coupled wind–vehicle–bridge vibration. They used a finite element model to simulate the dynamic response of the structure under the combined effect of wind load and vehicle load and concluded that the wind load caused fluctuations in the vibration displacement curves. Zhao et al. [14] analyzed the wind-induced vibration characteristics of a curved girder unilateral cable-stayed bridge (CBUSB) by using a cross-section model wind tunnel test and finite element simulation. They compared the results with those from laboratory tests performed on a bridge scaled down to 1:10 and plotted the relationship between wind speed and bridge mid-span displacement. The prediction of the dynamic response of bridge structures under strong wind conditions is often based on wind loads obtained through laboratory experiments. However, the actual wind loads can differ from the simulated values in these experiments, leading to differences between the predicted results and the actual dynamic response of the bridges. With the further development of bridge health monitoring systems, it has become possible to collect wind speed information and obtain wind load characteristics in real time. This allows the use of finite element models to predict the displacement response of bridges under actual wind loads, and by comparing the predicted results with the measured data, the accuracy of model predictions can be evaluated. FEM can be employed to establish virtual models of bridge structures, allowing for the analysis of the dynamic and static responses of all components in different load scenarios or with multiple load combinations. However, bridges are often subjected to complex loading conditions influenced by various factors and may be affected by noise [15]. With the development of bridge health monitoring systems and big data technologies, data-driven methods supported by machine learning (ML) can be used to predict the actual dynamic responses of bridge structures. Through assimilation and training with historical response data from numerous bridge structures, it is possible to develop predictive models. Methods such as Support Vector Machine (SVM) [16], Random Forests (RFs) [17], and neural networks [18] can be employed to predict the dynamic responses of bridge structures by learning the relationship between input features (such as wind speed, wind direction, and structural geometry) and output responses. In contrast to SVM and RF methods, neural networks excel in capturing intricate nonlinear connections, boasting adaptive learning capabilities that allow them to tailor predictions to diverse bridge structures and operating conditions [19,20]. Currently, data-driven prediction methods for bridge structural dynamics have mostly focused on Convolutional Neural Networks (CNNs) and Long Short-Term Memory (LSTM) [21,22]. Sun et al. [23] proposed a hierarchical convolutional neural network (HCNN) model for predicting bridge-bearing displacements using vehicle, wind, and temperature loads as input features. A case study of a cable-stayed bridge with a main span of 510 m and side spans of 215 m verified the effectiveness of this method in predicting bearing displacements, with an accuracy of more than 95.6%. Compared with the traditional CNN and encoder–decoder and U-Net models, this method shows superior performance. However, it should be noted that the range of bearing displacements in bridges is usually limited because the bearings are usually supported by pile foundations, which makes it difficult for large displacements to occur. The mid-span displacements of large-span bridge structures, especially under the influence of strong winds, may show significant variations and are susceptible to noise disturbances; therefore, more stable prediction methods are needed to take these factors into account. Deng et al. [24] introduced the concept of influence lines and proposed a vertical displacement prediction model for cable-stayed bridge decks based on recurrent neural networks (RNNs), using vehicle load and temperature as phase input variables. Wang et al. [25] proposed a deep learning-based framework for constructing LSTM and CNN models to predict the wind-induced transverse displacement response of cable-stayed bridge decks by considering wind speed as a model input. However, when considering bridge deck displacements, these prediction models need to take into account the significant differences in data characteristics, excitation sources, and load response mechanisms, and these models’ generalization ability is insufficient, affecting their prediction performance. Bayesian Neural Networks (BNNs), on the other hand, utilize Bayesian inference methods to update the posterior distribution of weights and biases based on observed data. This approach combines a priori knowledge with observed data, providing better resistance to noise and higher generalization ability [26].

Thanks to technological advancements like big data, the Internet of Things (IoT), and cloud platforms, digital twin (DT) technology has seen extensive adoption in the realm of structural health monitoring [27,28]. DT technology provides an efficient and accurate method for the improved monitoring of the health condition of bridge structures by allowing the creation of a virtual model that is consistent with the response of an actual bridge entity, combining a physically driven and data-driven dual-drive format [29].

Currently, research on dual-driven methods based on data and the FEM has received significant attention in the field of large-span bridge structural health monitoring. These methods entail refining the finite element model (FEM) with both measured and monitored data [30], alongside generating data samples through FEM simulations and variations in loading conditions. These data are subsequently integrated with machine learning algorithms [31,32]. This fusion-driven approach can more accurately predict and classify the condition and extent of structural damage, providing essential references for structural health monitoring and maintenance [33]. However, studies on dual-drive methods are more common in small- and medium-sized bridge structures, while studies on predicting the wind-driven dynamic response of large-span bridges using improved finite element models and machine learning-based dual-drive methods are limited. To address this research gap, this paper introduces a dual-driven approach that combines machine learning with a model-driven approach based on data obtained from the GNSS and Earth Observation for Structural Health Monitoring (GeoSHM) for predicting the dynamic response of a large-span bridge structure under strong winds, taking the FRB as a case study [34]. In contrast to the CNN neural network model, the BNN model employs a probabilistic approach to represent parameter uncertainty, thereby circumventing the need for maximum likelihood function optimization and effectively mitigating overfitting concerns. Furthermore, the BNN model exhibits rapid learning capabilities and demonstrates enhanced generalization performance, particularly when confronted with limited sample sizes. Regarding the FEM model, relying solely on wind tunnel experiments for predicting the dynamic response of bridge structures may yield inaccuracies due to disparities between the experimental setting and the actual environmental conditions in which the bridge is situated. To address this limitation, this research adopts a more robust approach by incorporating real-time wind loading. Specifically, the study leverages the GeoSHM system to capture real-time wind velocity data and subsequently incorporates these data into an optimized finite element model. By integrating real-world environmental data, this methodology enables more precise forecasts of the dynamic response exhibited by bridge structures. In this paper, two methods are proposed to predict the displacement and dynamic response of structures under strong winds, namely, a BNN model based on Bayesian inference and a finite element model method modified based on genetic algorithms and multi-objective optimization using response surface methodology, and we compare and analyze the characteristics of these two prediction methods. This analysis reveals the feasibility of a fusion-driven approach using both data-driven and physical model-driven methods in an integrated way and provides a useful reference and practical information for the application of DT technology in the field of bridge engineering.

After the introduction, Section 2 describes the FEM-driven method based on RSM correction, presents the methodology and steps of the data-driven method based on the BNN algorithm, and presents an evaluation methodology for both methods. In Section 3, the FRB is used as a case study to predict the dynamic response of a bridge structure using the two driving methods. In Section 4, we provide the prediction results of the two driving methods and use the data collected by the actual GeoSHM system as a reference to compare and analyze the accuracy, computational efficiency, system complexity, data requirements, interpretability, and prediction comprehensiveness of the two methods for predicting the dynamic response of a large-span bridge structure under strong winds. Section 5 includes the conclusions drawn from the analyses and an outlook on the integration of the dual-drive methods.

## 2. Methods

**Figure 1:**
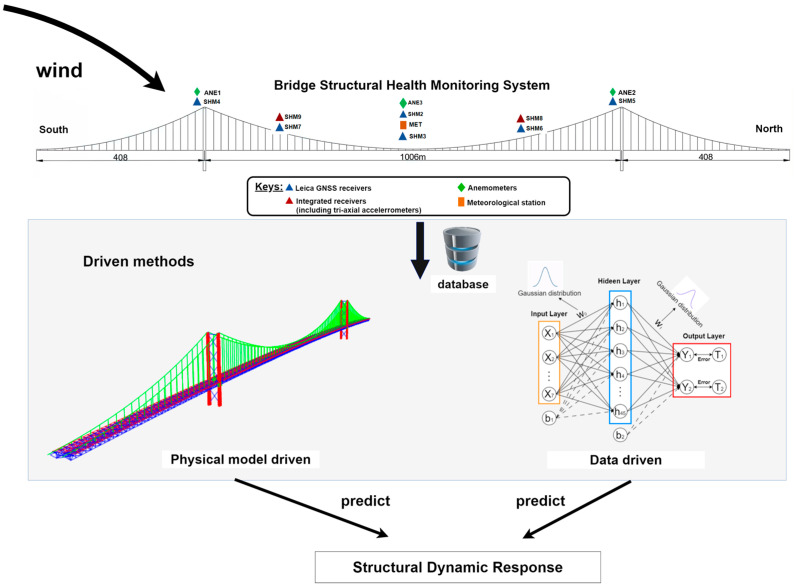
A framework for predicting the dynamic response of the two modes under the effect of strong winds.

The main objective of this paper is to compare and analyze the characteristics of two methods, i.e., the model-driven and data-driven methods, in the prediction of the dynamic response of large-span bridge structures under strong wind loading. The first part introduces the theory of FEA under strong wind effects and proposes the finite element correction method based on response surfaces. The second part introduces the principle of a BNN and the algorithm for predicting the displacement response of bridge structures, and the third part proposes a method for evaluating the performance of the two driving methods. The main contents of this section are shown in Figure 1, with reference to [20,35,36].

### 2.1. Finite Element Analysis Theory and Model Updating

#### 2.1.1. FEA Theory

When predicting the wind-induced dynamic response of bridge structures using finite element models, wind tunnel tests are often conducted to obtain the force states of the bridges under wind conditions. However, for in-service bridge structures, it is impractical to use wind tunnel tests to predict the real-time dynamic response of the bridge structure, as it requires significant computational costs. This paper utilizes the wind speed information collected by the structural health monitoring (SHM) system of the bridge structure and performs wind load time history analysis in the form of static wind loads to predict the quasi-real-time dynamic response of large-span bridge structures under strong wind conditions.

The Direct Integration Method (DIM) [37] is a commonly used FEM for determining the dynamic response of bridge structures. This method integrates the dynamic power balance equations of a structure in time to ascertain the displacement, velocity, and acceleration response of the structure for a given loading condition using the Hilber–Hughes–Taylor (HHT) algorithm [35]. The Direct Integration Method is an implicit time-integration method that combines a linearly weighted residual approach with a linear acceleration approach to provide greater numerical stability and accuracy.

The power balance equation of the HHT method can be expressed as follows [35]:

(1) $$\left(1+\alpha \right)M{\ddot{u}}_{n+1}+\left(\gamma +\alpha \right)C{\dot{u}}_{n+1}+\left(\beta +\alpha \right)Ku_{n+1}=F_{n+1}+F_{n}+\alpha \left(M{\ddot{u}}_{n}+C{\dot{u}}_{n}+Ku_{n}\right)$$

where M is the mass matrix, C is the damping matrix, K is the stiffness matrix, ${\ddot{u}}_{n+1}$ is the acceleration at the (*n* + 1)*th* time step, ${\ddot{u}}_{n}$ is the acceleration at the *nth* time step, ${\dot{u}}_{n+1}$ is the speed increment for the (*n* + 1)*th* time step, ${\dot{u}}_{n}$ is the speed increment for the *nth* time step, $u_{n+1}$ is the displacement at the (*n* + 1)*th* time step, $u_{n}$ is the displacement at the *nth* time step, $F_{n+1}$ is the external load for the (*n* + 1)*th* time step, $F_{n}$ is the external load for the *nth* time step, and α,β,γ are the control parameters of the HHT method.

This study only considers the external loads of dead load, temperature load, and static wind load. Therefore, the external load, F, of a structure can be expressed as

(2) $$F=F_{D}+F_{T}+F_{W},$$

where $F_{D}$ is the gravity loading, $F_{T}$ is the temperature loading, and $F_{W}$ is the static wind loading.

For large-span bridge structures, static wind loading is commonly dominant, and in most cases, the effect of static wind loading on the displacement response of a structure is greater than that of dynamic wind loading. To simplify the analysis, only the static wind load is considered when performing the displacement response analysis under long-term loading. $F_{W}$ includes the wind loading acting on the bridge structure in the longitudinal, X; transverse, Y; and vertical, Z, directions, which can be expressed as

(3) $$F_{W}=\left[\begin{array}{c} F_{X}\\ F_{Y}\\ F_{Z} \end{array}\right],$$

(4) $$F_{X}=\frac{1}{2}\rho {\cdot V}_{X}^{2}\cdot C_{d}\cdot A_{X},$$

(5) $$F_{Y}=\frac{1}{2}\rho {\cdot V}_{Y}^{2}\cdot C_{d}\cdot A_{Y},$$

(6) $$F_{Z}=\frac{1}{2}\rho {\cdot V}_{Z}^{2}\cdot C_{d}\cdot A_{Z},$$

where ρ is the air density; $V_{X}$ is the wind speed component in the X-direction; $V_{Y}$ is the wind speed component in the Y-direction; $V_{Z}$ is the wind speed component in the Z-direction; $C_{d}$ denotes the wind pressure coefficients in the X-, Y-, and Z-directions; $A_{X}$ is the wind-affected area in the X-direction; $A_{Y}$ is the wind-affected area in the Y-direction; and $A_{Z}$ is the wind-affected area in the Z-direction.

The assumptions made in the finite element model of a bridge can have an impact on the simulated results. These assumptions include the assumption of linear elasticity, rigid connection, material isotropy, and element discretization. All these assumptions can lead to differences between the actual state of a bridge under loading and the state simulated by the finite element model, thus affecting the accuracy of a model. The assumption of linear elasticity is reasonable within a small strain range but can cause nonlinear behavior when a material exceeds its linear elastic limit. This can result in a model’s inability to accurately predict the deformation and stress response of a material. For large-span bridges, under the influence of strong winds, the beam and plate structures are generally assumed to undergo small deformations, while the suspension elements may experience significant deformations. Therefore, it is necessary to consider the nonlinearity of the material used. Nonlinear material models, such as elastic–plastic models, are employed to handle this nonlinear behavior of suspension elements.

#### 2.1.2. FEM Update

Enhancing the model’s accuracy and reliability, accurately predicting structure behaviors and responses, and aligning the finite element model (FEM) more closely with real-world conditions necessitates corrections of the FEM. When correcting the finite element model of large-span bridge structures, conducting finite element simulations in practical operations can be time-consuming. Therefore, an efficient method is needed to estimate the output values of complex problems. Response Surface Methodology (RSM) [36] is a technique in which an approximate model of a complex system is constructed to estimate the output values, significantly reducing computational costs. This method can generate responses based on given inputs and be used to perform model prediction and optimization. When correcting a finite element model of a large-span bridge structure, numerous parameters and complex nonlinear relationships are involved. To address this, in this study, we employ a simulation-based evolutionary optimization algorithm known as the genetic algorithm (GA), which applies the principles of survival of the fittest to search for optimal solutions within a population. Additionally, due to the existence of multiple conflicting optimization objectives, such as structural stiffness, self-weight, and vibration performance, Multi-Objective Optimization (MOO) methods can be employed as they allow one to utilize multiple objective functions for optimization and find a balance and optimal solution among multiple objectives [38]. Therefore, in this paper, we propose a multi-objective optimization response surface methodology based on the genetic algorithm to correct the finite element model of large-span bridge structures.

RSM is a statistical and mathematical tool used for approximating functions. By establishing explicit approximate relationships between structural features and design variables, known as response surface modeling, RSM can replace finite element models for iterative computations. The mathematical expression of the response surface function is as follows [39]:

(7) $$y=f\left(x_{1},x_{2},\cdot \cdot \cdot x_{k}\right)+\varepsilon ,$$

where f is the mapping relationship between input and output variables; x is the input variable; k is the number of parameters; and ε is the statistical error.

The form of the response surface function has a significant impact on the accuracy of model correction. Therefore, it is important to select an appropriate function form based on the corresponding structural features. When performing finite element model correction for bridge structures, first-order polynomial or second-order polynomial function models are commonly considered. The first-order polynomial response surface function has fewer unknown coefficients, resulting in lower computational complexity, but it can only fit linear functions well, while the second-order polynomial response model offers higher accuracy in model correction. Hence, in this paper, a second-order polynomial function is chosen for regression in the response surface function.

MOO is a branch of operations research that has emerged as a new discipline in recent years. It focuses on the problem of optimizing multiple objective functions under certain constraints. A typical multi-objective optimization problem consists of multiple objective functions and a set of equality or inequality constraints, which can be mathematically described as follows [40]:

(8) $$\left\{\begin{array}{c} \underset{\min/\max}{F}(\vec{x})=(f_{1}(\vec{x}),f_{2}(\vec{x}),\cdots f_{n}(\vec{x}))\\ s.t.g_{i}(\vec{x})\leq 0,i=1,2,\cdots ,m,\vec{x}\in \Omega \end{array}\right.,$$

where $\vec{x}$ is a vector of decision variables, which, in finite element model optimization, constitutes the parameters to be corrected; $f_{1}(\vec{x}),f_{2}(\vec{x}),\cdots f_{n}(\vec{x})$ is the objective function, which, for finite element model correction, consists of minimizing the difference between the response surface function and the finite element prediction; s.t. denotes the constraints; $g_{i}(\vec{x})$ is the constraint function; and m is the number of constraints.

**Figure 2:**
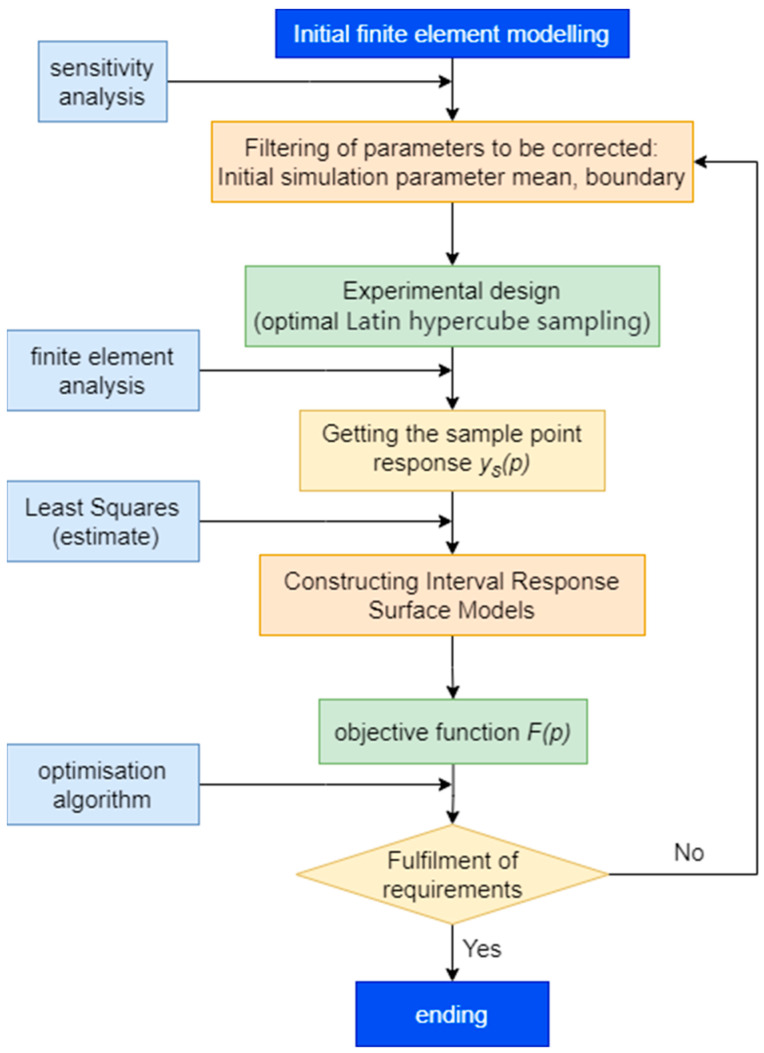
Finite element correction process.

Figure 2 shows the steps of FEM correction using the RSM method.

The steps to be followed are shown below:

1. Establish an initial FEM of a bridge;
2. Screen the parameters to be corrected and perform the initial fitting of the mean value of the parameters;
3. Conduct an experimental analysis (optimal Latin hypercube sampling (LHS));
4. Obtain the sample point response $y_{s}(p)$;
5. Construct the response surface model;
6. Determine the objective function F(p);
7. Optimize the parameters.

During the parameter-screening process in Step 2, typical material parameters for bridge structural components include the modulus of elasticity and density. These parameters directly affect the stiffness matrix K and mass matrix M of the members, which, in turn, have an impact on the self-oscillation frequencies of the bridge. Therefore, the elastic modulus and density of the main-span steel truss, side-span steel truss, main tower, and bridge deck are selected as the reference parameters for correction. The range of values for the initial parameters is based on actual engineering experience and design requirements.

Sensitivity analysis entails that when other parameters remain unchanged and a perturbation e (the variation range of bridge structural parameters is limited, usually within 10%; 5% was chosen in this paper) is set to the parameter *x*, when *y* becomes $y_{i}$, the local sensitivity of the parameter *x* can be approximated as follows:

(9) $$\delta =\frac{y_{xi+\varepsilon }-y_{xi}}{y_{xi}}\times 100\%,$$

In Step 3, experimental analyses are conducted to determine an appropriate set of sample points using the optimal LHS method [41], which guarantees a uniform distribution of sample points across the parameter space to thoroughly explore it. The locations of these sample points can be defined as follows:

(10) $$X_{ij}=\frac{{(r}_{i}+(j-1))}{k},$$

where $X_{ij}$ is the sample point location, *i* is the dimension of the sample parameter, k is the equally spaced interval that divides the parameter space, and j=1,2,...,k, $r_{i}$ denote the random numbers in each parameter dimension i. With this formula, a matrix of size k×n can be generated, where each row corresponds to a sample point and each column corresponds to a parameter dimension.

Frequency is one of the important dynamic characteristics of bridge structures, directly reflecting the inherent vibration frequency of the structure. By optimizing the finite element model of a bridge, aiming to achieve a close match between the calculated and measured frequencies, the reliability and accuracy of a model can be enhanced. Regarding Step 4, obtaining the response of the sample points involves performing model analysis on the initial finite element model to calculate the state of the model in its initial condition. The frequency response of the bridge structure was chosen as the objective *F*(*p*).

In Step 5, the parameter values from the sample data are formed into a design matrix X, and the corresponding self-oscillation frequency data are formed into a response vector Y. A regression analysis is performed using the least squares method to fit the extended design matrix X and the response vector Y, and the coefficients of the model are estimated. Then, the response surface model is constructed. The relationship between the parameters and the self-oscillation frequency is expressed as a quadratic polynomial fitting model. The response surface model formula is as follows:

(11) $$y=\beta_{0}+\beta_{1}x_{1}+\beta_{2}x_{2}+\ldots \;+\beta_{n}x_{n}+\beta_{11}x_{12}+\beta_{12}x_{1}x_{2}+\ldots +\beta_{\mathrm{nn}}x_{n2},$$

where y is the self-oscillation frequency, $x_{1},x_{2},\ldots ,x_{n}$ denote the parameters, and $\beta_{0},\beta_{1},\beta_{2},\ldots ,\beta_{n},\beta_{11},\beta_{12},\ldots ,\beta_{\mathrm{nn}}$ are the coefficients of the model.

The response surface models developed were evaluated to check the degree of fit and their reliability. The Coefficient of Determination ($R^{2}$) and root-mean-square error (RMSE) were used to assess the performance of the models.

In Step 6, the difference between the predicted value and the actual observed value is measured using the mean value of the relative error as the objective function *F*(*p*), where *F*(*p*) can be defined as the mean value of the relative error, denoted as

(12) $$F\left(p\right)=\frac{1}{n}\times \sum \left|\frac{\left(f_{exp}-f_{pred}\right)}{f_{exp}}\right|,$$

where $F\left(p\right)$ is the objective function, *n* is the number of sample points, $f_{exp}$ is the actual observed self-oscillation frequency, and $f_{pred}$ is the self-oscillation frequency predicted by the response surface model developed.

Ultimately, the GA and MOO algorithm is employed to seek the optimal parameter combination within the parameter space, aiming to minimize the objective function *F*(*p*). These optimized parameters are then input into the FEM to obtain a modified FEM.

### 2.2. BNN Theory and Predictions

#### 2.2.1. BNN Theory

Predicting the dynamic response of a structure based on the environment in which a bridge is located and the external loading is a regression problem. Therefore, the prediction requires a unique regression function value for the desired structural response, such as displacement, stress, strain, or other structural responses. This limitation determines the incompleteness of BNN in predicting structural responses. In Bayesian linear regression, we introduce a prior distribution to describe the uncertainty of the regression parameters. A Gaussian prior distribution is used as a prior for the regression parameters so that the posterior distribution can be estimated using the Bayesian inference methods.

The dynamic response of the bridge is set as the objective function *t*. Vehicle loading, wind loading, temperature, humidity, and other parameters are used as input characteristic parameters x. The objective function can be expressed as follows [42]:

(13) t=y(x,w)+ε,

where $x=(x_{1},x_{2},\ldots ,xD)T$. This is often simply known as linear regression. ε is a zero-mean Gaussian random variable with precision (inverse variance) $\sigma^{2}$.

This requires that we evaluate the predictive distribution, defined as

(14) $$p(t|x,t,\alpha ,\sigma^{2})=\int p(t|x,w,\sigma^{2})p(w|x,t,\alpha ,\sigma^{2})\;dw,$$

where t is the vector of target values from the training set. The conditional distribution $p(t|x,w,\sigma^{2})$ of the target variable is given by the following equation:

(15) $$p(t|x,w,\sigma^{2})=N\;(t|y(x,w),\sigma^{2}),$$

The posterior weight distribution is given by the following equation:

(16) $$p(w|x,t,\alpha ,\sigma^{2})=N\;(w|m_{N},S_{N}),$$

The mean and variance $m_{N},S_{N}$ of the sample weight $w_{i}$ (*i* = 1, …, *n*) can be determined using

(17) $${S_{N}}^{-1}={\sum_{0}}^{-1}+\frac{\Phi^{T}\Phi }{\sigma^{2}},$$

(18) $$m_{N}=S_{N}\left({\sum_{0}}^{-1}\mu_{0}+\frac{\Phi^{T}t}{\sigma^{2}}\right),$$

where Φ denotes the sigmoid activation function and is defined as $\Phi \left(x\right)=1/(1+e^{-x})$.

The first term in ${S_{N}}^{-1}$ represents the noise in the data, whereas the second term reflects the uncertainty associated with the weight parameters ***w***.

#### 2.2.2. BNN Training

The data set is divided into a training set and a test set. The training set is used to train the BNN model, while the test set is used to evaluate the model and make predictions.

Based on the input parameters, the first procedure is to obtain the H value of the hidden layer. After randomly generating the initial input weight $W_{0}$ and the offset $W_{1}$, the hidden layer H can be expressed as follows:

(19) $$X=W_{0}\times X_{0}+B,$$

(20) $$H=\frac{1}{1+e^{-X}}$$

where $X_{0}$ denotes the initial input.

The next procedure is the key for the BNN, and it iteratively determines the weight $W_{1}$ from the hidden layer to the output layer [42]. Two hyperparameters, a,β, are chosen to make predictions by marginalizing with respect to W. Hyperparameter a is a zero-mean prior isotropic Gaussian distribution, and the prior distribution of w is given by the following equation:

(21) $$p\left(w|a\right)\sim N(w|0,a^{-1}I),$$

The hyperparameter β is the precision of the noise of the target variable t, and the noise obeys a zero-mean Gaussian distribution and can be rewritten as

(22) $$p\left(t|x,w,\beta \right)\sim N(t|y\left(x,w\right),\beta^{-1}I),$$

A solution framework named empirical Bayes/evidence approximation is employed to estimate the hyperparameters, which are determined by maximizing the marginal likelihood function through integrating over the weight parameters w; hence, it is given as

(23) $$p\left(t|a,\beta \right)=\int p\left(t|w,\beta \right)p\left(w|a\right)dw,$$

Set parameter A as follows:

(24) $$A=aI+\beta \Phi^{T}\Phi ,$$

where Φ denotes the sigmoid activation function. The eigenvalues of A can be given as $a+\lambda_{i},$ and equations can be devised for maximizing the evidence function:

(25) $$a{m_{N}^{T}m}_{N}=M-a\sum_{i}\frac{1}{\lambda_{i}+a}=\Upsilon ,$$

(26) $$\Upsilon =\sum_{i}\frac{\lambda_{i}}{\lambda_{i}+a},$$

(27) $$a=\frac{\Upsilon }{{m_{N}^{T}m}_{N}},$$

Here, M is the number of training data, and $m_{N}$ is the mean of the Gaussian posterior distribution of p(w|t), which can be determined using Equation (17).

**Figure 3:**
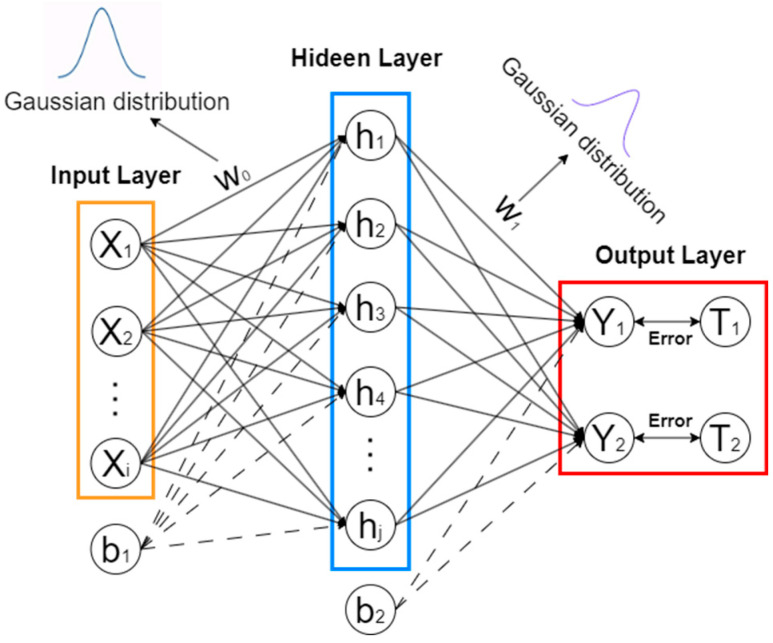
The training procedures for the BNN SHM algorithm.

BNN models address uncertainties in input data and nonlinear behavior by introducing probability distributions to represent parameter uncertainty. The uncertainty of BNN predictions can be quantified through output probability distributions, providing measures such as mean, variance, or confidence intervals. These uncertainty estimates enhance the reliability, robustness, and interpretability of a model’s predictions in real-world applications. A training diagram is shown in Figure 3. In the figure, *i* is the number of input parameters, and *j* is the number of hidden layers. It can be seen that the network architecture of the BNN is simple.

The first procedure entails making an initial choice a to find $m_{N}\;and\;\Upsilon$, which are given by Equations (17) and (25). These values are then used to re-estimate a using Equation (16). This process is repeated until $m_{N}$ converges. The procedure for obtaining the value of hyperparameter β is similar to the estimation of hyperparameter a, and it is described in detail in Chapter 3.5 in [33].

#### 2.2.3. BNN Prediction

When training has been successfully completed, a BNN model between the loading and environmental data and the dynamic response (lateral and vertical displacements) is devised. The model predicts the output using the loading data and environmental data as inputs (including traffic, wind and temperature, humidity, and pressure). Based on the predicted response matrix and the true response matrix, the loss value is calculated using Mean Squared Error (MSE), as well as the Coefficient of Determination ($R^{2}$) as the loss function.

(28) $$MSE=\sqrt{\frac{\sum_{n}^{1}{\left(y_{pred}-y_{actual}\right)}^{2}}{n}}$$

(29) $$R^{2}=1-(\frac{{SS}_{res}}{{SS}_{total}})$$

Here, $y_{pred}$ denotes the predicted value, $y_{actual}$ denotes the actual observation, n denotes the number of samples, and ∑ denotes the summation operation. ${SS}_{res}$ denotes the Sum of Squares of Residuals, $and\;{SS}_{total}$ denotes the Total Sum of Squares.

### 2.3. Comparison of FEM and BNN Bridge Structure Dynamic Response Prediction Results

**Figure 4:**
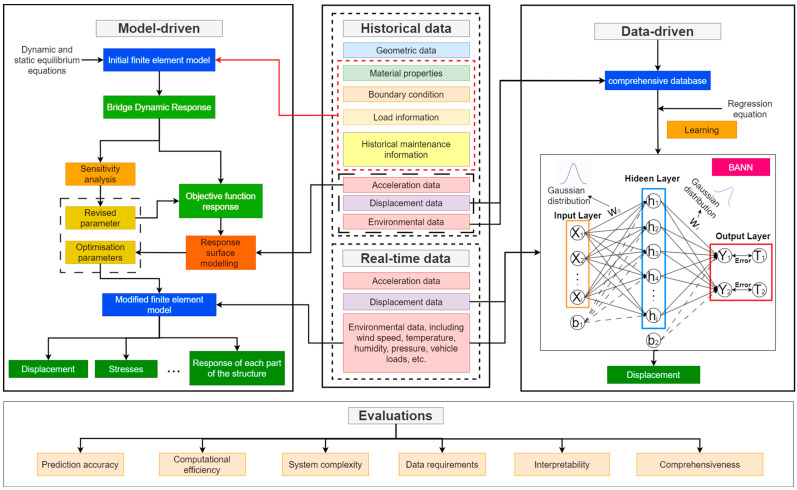
The process of predicting the structural dynamic response of a large-span bridge using two driving approaches, i.e., a model-driven approach based on FEA and a data-driven approach based on the BNN algorithms.

In investigating the prediction of large-span bridge structural dynamic responses through the two proposed methods in high-wind conditions, weather conditions characterized by strong winds should be selected for the corresponding experiment. In our study, a database was established using both historical and real-time data. Historical data include bridge geometry information, material properties, connection characteristics, maintenance records, and historical load information, in addition to environmental information and structural response data stored in the GeoSHM. Real-time data include real-time load information, environmental information, and structural responses collected by the GeoSHM. Figure 4 depicts the procedure for forecasting the structural dynamic response of a large-span bridge subjected to intense wind loading using two distinct driving methodologies: a model-driven approach based on FEA and a data-driven approach utilizing the BNN algorithm.

An FEM of the large-span bridge is established as a physical model, simulated based on known physical equations and structural parameters, and the FEM was corrected using the RSM. The collected load information, environmental information, and structural response are used for model training using the BNN-based data-driven approach. The data are divided into a training set and a test set. The model is trained using the training set, and then the predictive performance of the model is evaluated using the test set.

Based on the predicted results, the data-driven and physical model-driven methods are compared and analyzed. This comparison can be made in regard to the following aspects:

Prediction accuracy—Compare the accuracy of the two methods in predicting the structural displacement response of large-span bridges. Compare the prediction results using assessment metrics such as the RMSE and the $R^{2}$.

Computational efficiency—Compare the two methods in terms of computational speed and resource consumption, time taken for model training and prediction, and computational resource requirements.

System complexity—Compare the complexity and difficulty of the two methods in the process of model building and application, considering the difficulty of model parameter setting, model tuning, and implementation.

Data requirements—Compare the data requirements and availability of the two methods, including with respect to the quantity of data, the accuracy of the data, and the ease of data acquisition.

Response prediction types—Compare the applicability and accuracy of the two methods in dealing with different types of response predictions (e.g., displacements, stresses, model parameters, etc.).

Interpretability—Compare the ability of the two methods to interpret and understand the results, considering the interpretability of the model outputs, the interpretation of the predicted results, and the ability to analyze the prediction errors.

## 3. Case Study

### 3.1. Overview of the Forth Road Bridge and the GeoSHM Sensor System

**Figure 5:**
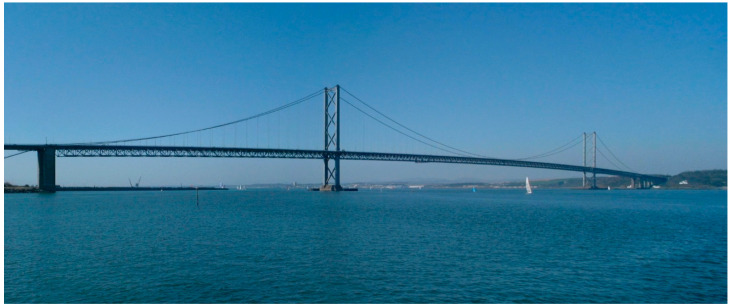
The FRB in Edinburgh, U.K. (Stuart Halliday/CC-BY-3.0).

The Forth Road Bridge (FRB) in the United Kingdom is a representative example of a long-span bridge and has been widely studied as a case for the early deployment of structural health monitoring systems. The FRB spans the River Forth, connecting Edinburgh in South Queensferry with Fife in North Queensferry (Figure 5). The central main span of the bridge is 1006 m long. Each of the two side spans is 408 m long; the northern approach is 257 m long, and the southern approach is 438 m long, amounting to a total length of 2512 m. The deck of the main span consists of orthotropic anisotropic steel plates, and the decks of the side spans consist of composite concrete slabs on steel girders.

The FRB was used as a testbed bridge for the GeoSHM Feasibility Study (FS) and Demonstration (Demo) project, both mainly supported by the European Space Agency (ESA) and led by the University of Nottingham (FS) and UbiPOS UK Ltd. (located in London, UK), to evaluate the integrated application of GNSS and Earth Observation (EO) technologies to Long-Bridge SHM using advanced sensor systems [43].

**Figure 6:**
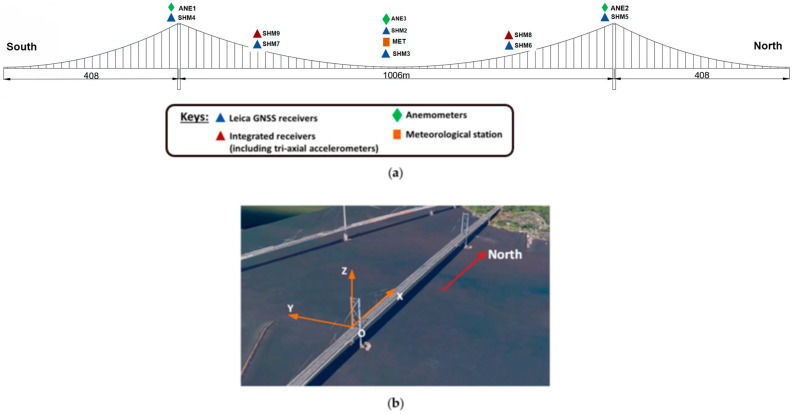
(**a**) The current GeoSHM sensor system and (**b**) the definition of the FRB coordinate system.

The comprehensive GeoSHM system comprises processing and monitoring modules, a bridge structure evaluation and early warning module, and a data management module. The current architecture of the GeoSHM sensor system for monitoring the FRB is shown in Figure 6 [44]. The data-processing and monitoring modules are deployed in the main server of GeoSHM, including preprocessing and post-processing modules. The primary task of the preprocessing unit is to process data from various sensors using GPS time synchronization, identify and remove outliers, and transform the raw sensor data into a format suitable for monitoring analysis. The output of the preprocessing unit is transmitted to the post-processing unit, which evaluates the 10 min statistical average values of features related to bridge deformation, external loads, and environmental influences [44]. The response of the main span is measured by three pairs of GNSS receivers. One pair is installed on the two sides of the mid-span point, while the other two pairs are installed at the navigational points (i.e., at the four quarter-span points) [43]. Two further monitoring points are installed atop the supporting towers. The wind measurements are collected using three Gill WindMaster 3D sonic anemometers placed at the west site of the mid-span and on the top of the two main towers. Other environmental parameters such as air temperature, pressure, and humidity are also obtained by a Gill MetPak meteorological station installed at the east site of the mid-span point. Furthermore, two integrated monitoring sensors called GeoSHM-Pro that include tri-axial accelerometers and multi-mode GNSS receivers are installed at two west-side quarter-span points to measure both the deformation and vibration of the bridge. More details on the GeoSHM-Pro sensor system can be found in [43,44].

### 3.2. Field Measurements and Wind Characteristics

The FRB, situated in the east-central region of Scotland, stretches across the Firth of Forth. It is situated in a temperate marine climate influenced by the warm currents of the North Atlantic, resulting in warm and humid weather conditions. The Firth of Forth is one of the windier parts of the U.K., being relatively close to the track of Atlantic depressions. The strongest winds are associated with the passage of deep areas of low pressure close to or across the U.K. The frequency and strength of these depressions are greatest in the winter half of the year, especially from December to February, and this is when mean speeds and gusts (with short-duration peak values) are high. The coast of the Firth of Forth has higher wind speeds and greater wind duration than most other parts of east Central Scotland.

The monitoring data used in this study correspond to the period from 2 February 2023 to 27 February 2023, when most of the seasonal storms occur. Gill WindMaster 3D sonic anemometers were used to record wind speed data and analyze them to ascertain the average wind speed characteristics.

Before analyzing the GeoSHM system, all the data collected by GNSS receivers and the anemometer installed on the FRB needed to be synchronized. Although the embedded software automatically transformed the time into uniform computer time and the coordinate system into a bridge coordinate system (BCS), the accuracy of each device is different, resulting in the time nodes of each record per second being slightly different. To achieve precise time synchronization, all sensors were time-stamped with the output of a hardware time server that is precisely aligned to GNSS time.

During the wind load analysis, the average of the three wind speed sensors was taken: 10 min was taken as a basic interval.

**Figure 7:**
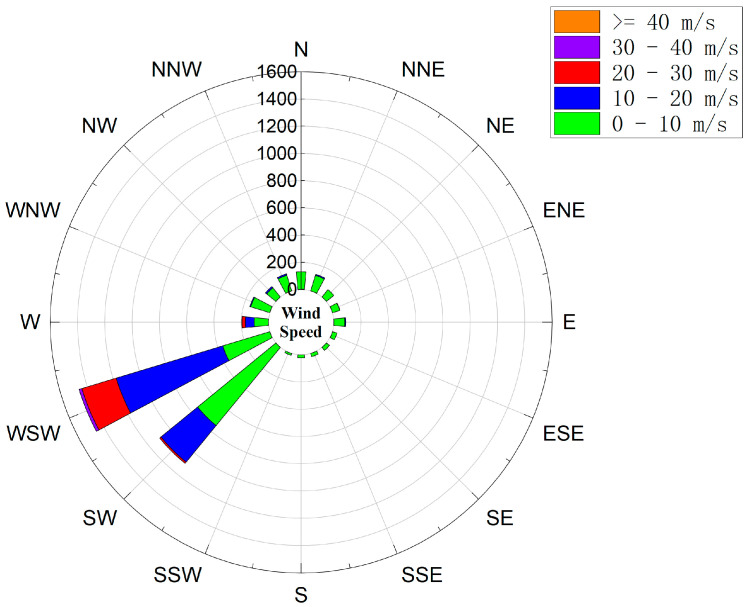
Wind rose diagram of maximum 10 min mean wind speeds.

Figure 7 shows that wind speed changes significantly with direction. In storms, the wind direction is usually from southwest (SW) to northeast (NE). The wind speeds are mostly between 10 m/s and 20 m/s. Wind speeds between 20 m/s and 30 m/s frequently occur, and wind speeds greater than 40 m/s can occur. The direction is roughly perpendicular to the bridge.

**Figure 8:**
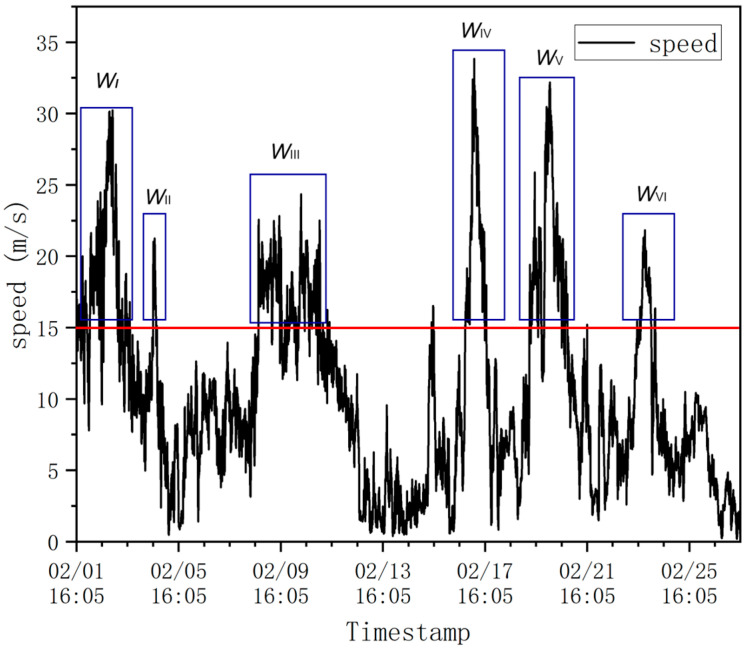
Wind speed at the Forth Road Bridge in February 2023.

According to the definition provided by the World Meteorological Organization (WMO), a strong wind (Strong) is a wind force of 6 to 7 on the Beaufort scale, i.e., 41 to 62 km per hour, equivalent to 22 to 33 knots per hour or 11 to 17 m per second. As can be seen from Figure 8, using the limit of 11 m per second to classify strong winds, denoted by the red line, there were six periods of strong winds in February, namely, 02.01–02.02, 02.04, 02.08–02.10, 02.16, 02.18–02.19, and 02.23, with the strong winds being labeled (in order) as ${Wind}_{I}$, ${Wind}_{II}$, ${Wind}_{III}$, ${Wind}_{IV}$, ${Wind}_{IV}$, and ${Wind}_{VI}$.

### 3.3. Physical Model

Compared to data-driven machine learning methods, finite element analysis often has a greater time cost, and finite element model optimization is a necessary step to ensure the accuracy of finite element models. RSM can efficiently estimate the output values of complex finite element models, significantly reduce computational cost, and thus improve the efficiency of finite element model prediction. In this study, a MOO algorithm and GA based on RSM is proposed. In this method, the GA is used to solve the nonlinear relationship between the parameters to be improved, determine the complex dynamic response of extra-large-span bridges, and find the optimal solution among the parameters. It should be noted that MOO using GA often results in multiple sets of optimal solutions, and it is necessary to compare multiple sets of test results to obtain the optimal parameters that minimize the difference between the finite-element-predicted structural response and the calculated value of the response surface function.

#### 3.3.1. Building a Finite Element Model

**Figure 9:**
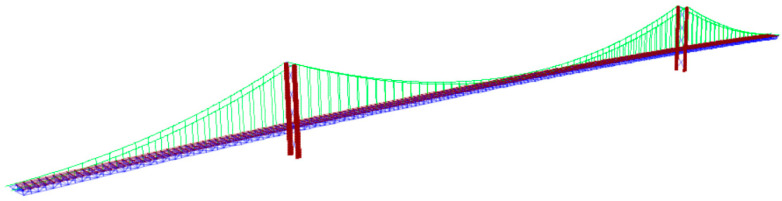
Sap2000 FEM schematic diagram of the FRB.

In this study, we used SAP2000 24 software to establish a spatial FEM of the FRB. The materials and unit types of each structural part are shown in Table 1. The resulting FEM of the FRB is shown in Figure 9.

**Figure 10:**
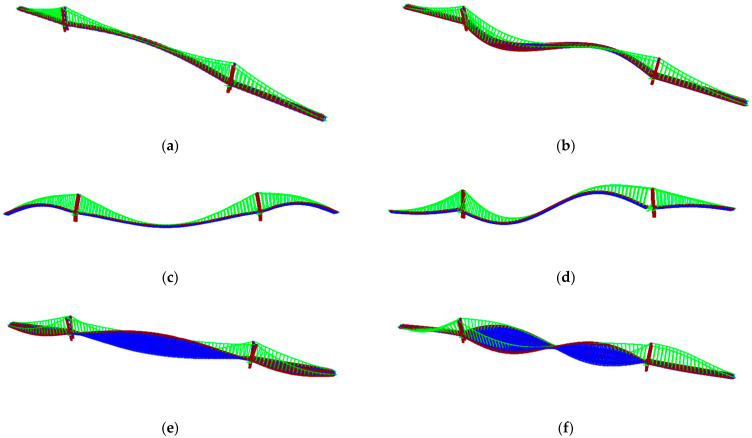
Initial analysis of the target frequency and formation of the FRB: (**a**) horizontal first-order formation; (**b**) horizontal second-order formation; (**c**) vertical first-order formation; (**d**) vertical second-order formation; (**e**) reversal of the first-order formation; (**f**) reversal of the second-order formation.

The subspace iteration method was used to calculate the dynamic characteristics of the initial FEM of the FRB. Figure 10 shows the first three vertical mode shapes, the first two torsional mode shapes, and the first two transverse mode shapes of the FRB.

#### 3.3.2. Construction of the Response Surface Model

The parameters of the FRB to be adjusted were identified, with mass and stiffness being commonly recognized as primary factors governing its dynamic properties. The modulus of elasticity and capacity of the main tower, deck, main girder, and suspension cables were chosen as the parameters to be corrected.

**Figure 11:**
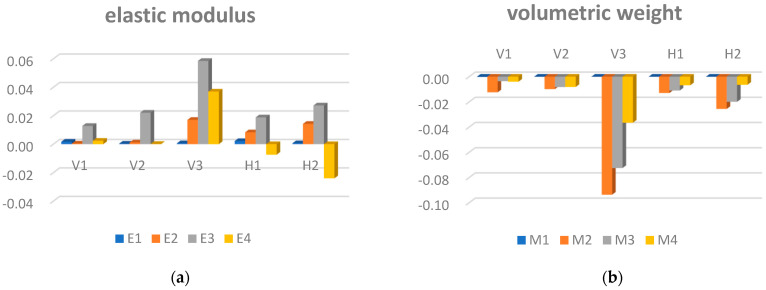
The sensitivity analysis results. (**a**) Sensitivity analysis of the modulus−of−elasticity values of different components; (**b**) sensitivity analysis of different component densities.

Local sensitivity analysis was performed on the identified model parameters slated for correction, and the results of this analysis are presented in Figure 11. Notably, the modulus of elasticity and the weight of the bridge deck, main girders, and suspension cables exhibit higher sensitivity. These six variables were consequently selected as parameters for correction when constructing the response surface model and denoted as $M_{1}$, $E_{1}$, $M_{2}$, $E_{2}$, $M_{3}$, and $E_{3}$.

The interval response surface model used in this paper is a second-order polynomial. For the six-parameter correction, the number of tests was not less than 13. For suspension bridge structures, due to the different degrees of influence of each parameter perturbation on the modes in the three directions, the order of appearance of the modes is often inconsistent with the test results when performing finite element calculations. In order to ensure the reliability of the calculation results, it is necessary to compare the modal shapes for each calculated modal frequency after simply setting up the loop and extracting the analysis results. Based on the above considerations, 21 sets of test samples were designed using the optimal LHS in order to save computational costs. Based on the tuning process of the initial FEM, the range of parameters to be corrected was determined, as shown in Table 2.

Least squares regression was performed using the multiple nonlinear regression function. Before fitting, the test set samples needed to be normalized. The random function can be used to generate a [0, 1] random vector to select the initial values of the parameters. We fit a regression curve between the adjusted parameters and the frequency values obtained from finite element calculations as the response surface. $R^{2}$ and RMSE were used to test the fitting accuracy of the interval response surface. The test results are shown in Table 3. It can be gleaned that the regression accuracy of the surrogate model for each modal frequency is high, and this model can be used to replace the FEM in subsequent calculations.

#### 3.3.3. Correction Finite Element Model

The natural frequency of the bridge was used as the objective function for model correction. The FRB 1 h span mid-acceleration data were selected. In order to improve computational efficiency, the Decimate function was first used to filter and resample the sample signal with a sampling rate of 5 Hz. The acceleration signal was processed using the Hilbert–Huang Transform (HHT) method to determine the bridge self-oscillation frequency. According to the methodology presented in Section 2.1.2, the optimal solution for the parameters to be modified was obtained using a genetic algorithm and a multi-objective optimization method. The initial population for the genetic algorithm was set as the initial values of the parameters, and the population size was determined within the range of variation for each parameter. The objective function for the multi-objective optimization aimed to minimize the difference between the response surface function and the frequency values predicted by the finite element analysis. Through the utilization of the genetic algorithm, multiple sets of optimal solutions were generated. The variance of the response surface functions, constructed based on each set of optimal solutions, and the frequency values calculated by the finite element model were computed. The set of optimal solutions with the smallest variance was then selected as the input parameters for the modification of the finite element model.

The wind speed data collected by the GeoSHM system are used to calculate the wind load functions in various directions according to Formulas (3)–(6). These wind load functions are then applied to the modified finite element model. Wind load time history analysis is conducted to calculate the dynamic response of the FRB (possibly referring to a specific component of the bridge structure) under the wind load time history function. These responses include stresses and strains of various components of the bridge structure, as well as the mode shapes, frequencies, and displacement values of the overall structure.

### 3.4. BNN Prediction

The data-driven method proposed in this paper is based on a Bayesian Neural Network (BNN), which is a powerful machine learning approach. Machine learning is used to discover and model the underlying relationships within data, and it requires a substantial number of data for effective training. By combining Bayesian inference with regression models, a BNN constructs a flexible and adaptable black-box model capable of capturing complex nonlinear relationships between input and output variables. The BNN model typically comprises multiple layers, including an input layer, hidden layers, and an output layer, enabling it to learn and represent intricate patterns in the data.

The goal of BNN prediction is to determine the regression relationship between loading, environmental effects, and structural displacement response output, and the regression model is developed with vehicle load, wind speed, temperature, humidity, and pressure as independent variables and structural displacement response as a dependent variable. Table 4 lists the input and output data used in this paper.

The sensor information is listed in Table 5.

Data collected by the GeoSHM sensors in February 2023 were used for analysis. The data from 1 to 20 February were used as a training set, and the data from 21 to 27 February were used as a test set to predict the Y- and Z-direction displacement responses in the bridge span. The BNN model was trained using the data collected from the GeoSHM system, including time, wind speed, wind direction, temperature, humidity, and pressure from 1st February to 20th February, and the GNSS sensor measurements of the mid-span displacement of the FRB were used as the target variable. The trained model was then used to predict the mid-span displacement response of the FRB under environmental loads from 1st February to 27th February.

Due to the lack of traffic data, the time in one day and day of the year were adopted to estimate the traffic loading according to traffic statistics. However, this estimation method can lead to inaccurate predictions of structural displacements caused by vehicle loads.

## 4. Results

### 4.1. Displacement Observed via GNSS

**Figure 12:**
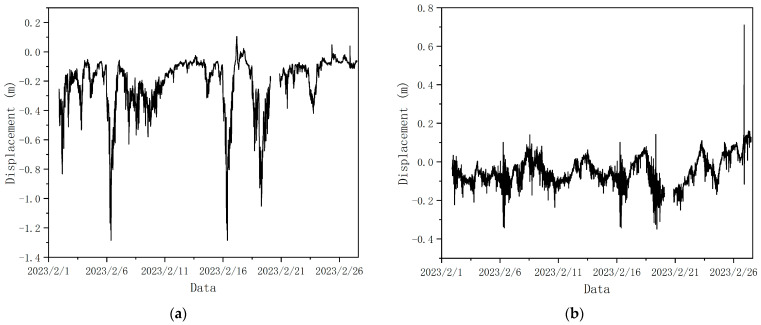
Displacement values in both directions at the central position of the bridge span in February 2023. (**a**) Y-direction displacement; (**b**) Z-direction displacement.

Bridge structures will exhibit abnormal structural behavior when their serviceability and structural integrity are compromised. The mid-span displacement of a large-span suspension bridge reflects the overall performance of the bridge and is an important indicator for assessing its structural condition [45]. The mid-span displacement of a bridge, therefore, provides vital information on the health conditions of the structure under the action of strong winds. The displacement data of the GeoSHM system were measured by GNSS and transmitted to the UbiPOS’ GeoSHM server located in London for processing, storage, analysis, and visualization. The displacement values collected by the sensors SHM2 and SHM3 of the main girder arranged in the middle of the span were used as output parameters to record the GNSS monitoring data in February 2023. The strong wind action mainly affects the Y- and Z-directions of the bridge; therefore, only the mid-span displacement values in the Y- and Z-directions predicted via BNN and FEM were analyzed. Firstly, the obtained GNSS was preprocessed with a data-sampling frequency of 10 HZ, and the data of SHM2 and SHM3 were aligned, as shown in Figure 12. The data for February are from 1 February 2023 21:40 to 27 February 2023 12:00, and in the middle of this set, the Y and Z displacement values of 20 February 2023 2:20–20 February 2023 20:50 are missing. The average value of the two sensors SHM2 and SHM3 was taken as the displacement value in the middle of the main span. Figure 12 shows the values of displacements in both directions at the mid-span location of the bridge for February 2023.

As can be seen in Figure 12, under the action of six strong winds, the Y-direction displacement at the main girder is larger, and the two largest displacements are −1.286 m and −1.286 m under the action of two strong winds, ${Wind}_{III}$ and ${Wind}_{IV}$, respectively. Furthermore, the value of Y-direction displacement is positively correlated with the square value of the magnitude of the wind speed, and the value of the Z-direction displacement in the span of the main girder is between −0.375 m and 0.145 m; the two largest displacements were also recorded under the action of ${Wind}_{III}$ and ${Wind}_{IV}$, amounting to −0.343 m and −0.350 m. It can be seen that the strong winds have a great influence on the structural displacements of large-span bridges.

### 4.2. Displacement Prediction of the BNN Algorithm and FE Model

In order to compare and analyze the characteristics of the two driving modes, the same data set was used to predict the structural response, and FEA was used to conduct a time course analysis from 21 February to 27 February.

Regarding the comparative analysis of the computational efficiency of the two driving methods, both the FEA and Bayesian predictions were made on a desktop computer running the Windows 11 operating system with an AMD Ryzen 7 5800H (with a Radeon graphics processor) and a 3.2 GHz CPU (with 16 GB of RAM). The FEA took approximately 5 h to complete the calculations, while Bayesian prediction took about 15 s. This indicates that the Bayesian prediction method has a clear advantage in terms of time efficiency, providing a fast and effective option for structural response prediction.

**Figure 13:**
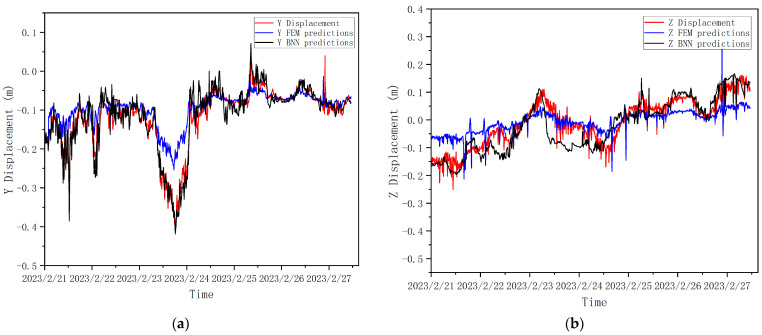
Prediction displacement of FE model and BNN. (**a**) Y-direction displacement; (**b**) Z-direction displacement.

As can be seen from Figure 13, the overall trends of the bridge mid-span displacements predicted via the FEM and BNN are consistent with the actual response. For the Y-direction displacement response (see Figure 12a), the displacement curves predicted using the FEM are consistent with the peaks of the actual response curves, and the fluctuation trends are similar, indicating that the prediction model was more capable of capturing the response characteristics of the bridge structure. However, there is a certain difference between the Y-direction displacement value predicted using the FEM and the actual displacement value, and the predicted value is smaller than the actual response value, which is due to the fact that the actual Y-direction displacement response of the bridge is affected by the wind load effect, the vehicle load effect, and the coupling effect of multiple loads. This difference is more significant during strong wind action (23 February) because the interaction between wind and bridge structure becomes more significant under strong wind action, and the dynamic action of wind vibrates the bridge structure, leading to the displacement response. When the wind load reaches a certain threshold, this interaction may cause a sudden large displacement, and the FEA currently does not consider the large displacement phenomenon. The shape of the displacement response curve predicted by the BNN and the actual corresponding displacement curve basically coincide, and the predicted value fluctuates up and down with a small amplitude above and below the actual response value, so the prediction results are more accurate compared with those of the FEM.

For the Z-direction displacement response (see Figure 12b), the displacement curves predicted using the FEM are similar to the general direction of the actual response curves, but there is a sudden change in the region with a larger wind load. This is because the Z-direction displacement is mainly affected by temperature, self-weight, and vehicle loading at times of small wind loads, and the vertical displacement in the bridge span is mainly affected by wind loads and changes with wind speed at times of large wind loads. However, in the actual bridge structure, the effects of vehicle load, temperature load, and coupling on the vertical displacement of the bridge will be greater than the effects of wind load, so there would not be any sudden changes. For vertical displacement, the displacement value predicted using the FEM is smaller than or equal to the actual response value, and the prediction results are conservative, which is due to the fact that the effect of temperature was not taken into account. The BNN-predicted response curves are still distributed within a certain range of the actual corresponding ones. However, the prediction results are not satisfactory for some time points, such as during the strong wind action on the 23rd–24th of February 2023. This is because the main influencing factors of vertical displacement are temperature loading and vehicle loading, while the bridge eigenvalues extracted by the BNN did not include accurate vehicle loading, and the training set data points are insufficient; thus, the model’s prediction effect is not sufficiently accurate.

### 4.3. Comparison of the Two Drive Modes

As shown in Section 4.2 in the accuracy analysis of displacement response prediction, the BNN is more accurate than the FEM prediction. In this study, we utilized RMSE and $R^{2}$ as evaluation metrics for model assessment. RMSE measures the average error between predicted and observed values, while $R^{2}$ quantifies the proportion of variance explained by a model’s predictions. These metrics were chosen due to their common usage and interpretability. Other potential evaluation metrics, such as MAE and confidence intervals, were considered but not included in this study. Table 6 shows the RMSE and $R^{2}$ predicted by the two models. The results indicate that the BNN achieves higher accuracy than the FEM in predicting displacements in both directions.

The computation time of the BNN is only 20 s, while the FEM takes 5 h. This highlights the higher computational efficiency of BNN for predicting displacement response in this specific problem. BNN’s simpler network structure and faster computations make it more efficient than FEM, which involves time-consuming processes such as cell discretization, matrix assembly, and solving large systems of linear equations.

FEM and BNN have different requirements for data quality and diversity. To develop an FEM, accurate knowledge of the geometry and material properties of the analyzed structure is required, along with specific loading conditions for strong winds. The BNN model relies on a set of training data with known displacement responses, including input characteristics and corresponding responses. Sufficient and diverse training data are crucial for the BNN model’s accuracy and generalization. Data quality issues and diversity affect the model’s performance, while incorporating feature engineering, such as wind-induced vibration information, can enhance predictions for strong wind-induced displacement responses.

FEM involves complex modeling of a structure into cells, considering factors like geometry, material properties, and loading conditions. FEM’s higher interpretability stems from its analysis of cell parameters to comprehend displacement mechanisms based on physical principles. However, the high complexity of the model also contributes to the lower computational efficiency of FEM. In contrast, BNN is a simpler three-layer neural network regression model with fewer network layers and a simpler structure. It is a black-box model, making it less interpretable. When using the BNN model to predict the dynamic response of bridge structures, if there is a significant difference or change in the trajectory direction between the predicted values and the actual values, it may indicate that the bridge structure has undergone certain changes or that there are abnormal conditions. In such cases, practitioners in the field of structural engineering should conduct inspections and maintenance of the relevant bridge structure.

The FEM used in FRB can predict not only the mid-span displacements in both the X and Y directions but also the overall response of FRB and the response of structural components under strong wind effects, such as torsion, deformation, local point displacements, and stresses. In contrast, the BNN model predicts the dynamic response of bridges solely as a regression analysis problem, limited to predicting displacement responses based on input wind loads and other engineered features. Furthermore, the FEM is a universal structural analysis method applicable to various types of bridge structures. However, the scalability of BNN models may have certain limitations. The focus of this study was on predicting the re-response of large-span suspension bridges under strong wind action. Wind loads and temperature loads have high sensitivity to the mid-span displacements of bridge structures, which are key engineering characteristics. Therefore, they exhibit suitable performance in predicting the responses of bridge structures under these conditions. However, to effectively utilize the BNN algorithm for predicting the dynamic response of other types of bridge structures or building structures, appropriate modifications and adjustments are required to accommodate the specific characteristics and response patterns of these structures.

The characteristics of the two model predictions are shown in Table 7.

## 5. Conclusions

In this study, we proposed and compared two different approaches, the Finite Element Method (FEM) and the Bayesian Neural Network (BNN), for predicting the dynamic responses of a large-span bridge under strong wind action.

First, we developed a response surface method based on a genetic algorithm for multi-objective optimization to refine the Finite Element Method (FEM). By utilizing wind speed information collected from the structural health monitoring (SHM) system of a large-span bridge, wind speed time history analysis was performed on the FEM model, successfully predicting the structural dynamic response of the bridge under strong wind action. The root-mean-square error (RMSE) values for the predicted model displacements in the transverse and vertical directions of the bridge (i.e., the FRB) were 0.0471 and 0.0514, respectively, with $R^{2}$ values of 0.6167 and 0.6283.

Furthermore, we used a Bayesian Neural Network (BNN) algorithm based on Bayesian inference theory to address the uncertainties and nonlinear behavior of input data. By analyzing the nonlinear relationship between environmental loads and the displacement response of the bridge, the BNN algorithm accurately predicted the displacement response of the bridge structure under strong wind action and demonstrated high generalization capabilities. The BNN model achieved RMSE values of 0.0232 and 0.0464 and R2 values of 0.9073 and 0.7969 for the transverse and vertical displacement predictions for the FRB, respectively. The transverse displacement prediction showed suitable results, as wind load is the main factor causing transverse displacement and is an important characteristic of large-span bridge structures, allowing for the extraction of more pronounced nonlinear features.

In conclusion, it can be stated that the Bayesian Neural Network (BNN) surpasses the Finite Element Method (FEM) in terms of accuracy and computational efficiency when predicting bridge displacement responses. The BNN demonstrates superior accuracy in predicting displacements in both directions and achieves a significantly faster computation time of only 20 s compared to FEM’s 5 h. This highlights the advantage of BNN’s regression-based approach and streamlined network structure, which contribute to its enhanced computational speed. However, FEM offers greater interpretability due to its reliance on physical principles, enabling a comprehensive analysis of structural responses under wind loading, including the prediction of stresses, strains, displacements, and natural frequencies at various locations. On the other hand, the BNN, being a black-box model based on neural networks, lacks interpretability and faces challenges in directly explaining the physical mechanisms of displacement response. Additionally, FEM necessitates accurate knowledge of geometry, material properties, and loading conditions, while BNN relies on a diverse set of training data for accurate predictions. Consequently, the selection between these methods depends on the specific analysis requirements, with FEM providing comprehensive analyses and predictions for bridge structures, while BNN offers higher accuracy and computational efficiency at the expense of interpretability and a narrower scope of prediction.

## Figures and Tables

**Table 1:** Materials and unit types of structural parts of the FRB.

Structural	Materials	Elastic Modulus (Pa)	Volumetric Weight (kg/m^3^)	Poisson’s Ratio
Girder	Steel	2.000 × 10^8^	76.9729	0.3
Main-span deck	Steel	2.100 × 10^8^	56.34	0.3
Side-span deck	Concrete	2.4855578 × 10^7^	23.563	0.2
Tower	Steel	2.000 × 10^8^	76.9729	0.3
Suspension cable	Steel wire	1.931 × 10^12^	78.075	0.3

**Table 2:** Parameter range to be corrected.

Parameter	$M_{1}$	$E_{1}$ **(×10^7^)**	$M_{2}$	$E_{2}$ **(×10^8^)**	$M_{3}$	$E_{3}$ **(×10^12^)**
Upper limit	26.610	2.950	85.460	2.300	84.110	2.100
Lower limit	18.870	2.000	65.490	1.700	72.040	1.770
Initial value	23.563	2.485	76.973	2.000	78.075	1.931

**Table 3:** Response surface model accuracy test.

	H1	V1	H2	V2	V3
$R^{2}$	0.9498	0.9911	0.9174	0.9520	0.9533
RMSM	0.0015	0.0019	0.2811	0.0053	0.0183

**Table 4:** The loading and responses of the BNN model.

Input/Loading		Output/Responses
Traffic	GPS week Seconds within a week	Lateral and heaving displacement
	Day of the year	
Environment	Normal component of wind speed	
	Pressure Temperature Humidity	

**Table 5:** The data acquisition information of the sensors installed on the FRB.

Sensor	Rate (Hz)	Manufacturer	Comments
GNSS	10	Leica/UbiPOS	3D deformation (X, Y, and Z)
Anemometer	10	Gill	Wind direction and speed
Meteorometer	1	Gill	Temperature, humidity, and pressure

**Table 6:** FEM and BNN prediction accuracy metrics.

	FEM Y	FEM Z	BNN Y	BNN Z
RMSE	0.0471	0.0514	0.0232	0.0464
$R^{2}$	0.6167	0.6283	0.9073	0.7969

**Table 7:** Characteristics of the two forecasting models.

Drive Modes	Predictive Accuracy	Computational Efficiency	Data Requirements	System Complexity	Interpretability	Comprehensiveness
**BNN**	high	fast	large number of data	low	low	single objective function
**FEM**	low	slow	accurate bridge geometry, material properties, connection characteristics	high	high	displacement response, stress response, vibration response, dynamic response, damage analysis of structural elements

## Data Availability

Data are not available due to restrictions regarding privacy or ethics.
